# Supplementary figures and images for: Internal consistency reliability, construct validity, and item response characteristics of the Kessler 6 scale among hospital nurses in Vietnam
Source: PLoS One. 2020 May 21;15(5):e0233119. doi: 10.1371/journal.pone.0233119 (PMC7241835; doi:10.1371/journal.pone.0233119)

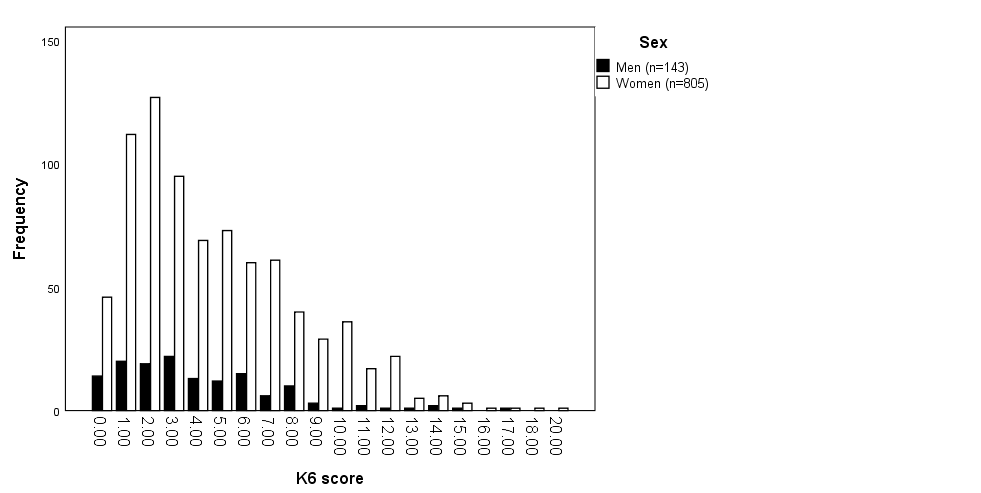

Supplement: S1 Appendix — (TIF) [file pone.0233119.s001.tif]
