# Supplementary material for: Internal consistency reliability, construct validity, and item response characteristics of the Kessler 6 scale among hospital nurses in Vietnam
Source: PLoS One. 2020 May 21;15(5):e0233119. doi: 10.1371/journal.pone.0233119 (PMC7241835; doi:10.1371/journal.pone.0233119)
Supplement: Attachment — Submitted filename: PlosOne-Response to Reviewers.docx [file pone.0233119.s003.docx]

Response to Reviewers (PONE-D-19-34231R1)

No comment from reviewers to this manuscript.
